# Plasma myeloperoxidase-conjugated DNA level predicts outcomes and organ dysfunction in patients with septic shock

**DOI:** 10.1186/s13054-018-2109-7

**Published:** 2018-07-13

**Authors:** Yuki Maruchi, Masanobu Tsuda, Hisatake Mori, Nobuyoshi Takenaka, Takayoshi Gocho, Muhammad A. Huq, Naoshi Takeyama

**Affiliations:** Department of Emergency and Critical Care Medicine, Aichi Medical University, Nagakute, Aichi 480-1195 Japan

**Keywords:** Neutrophil extracellular traps, Septic shock, Cell-free DNA, Myeloperoxidase, Neutrophil elastase, SOFA score, DIC

## Abstract

**Background:**

Recent studies have suggested that excessive formation of neutrophil extracellular traps (NETs) plays a critical role in the pathogenesis of sepsis. Although elevation of the plasma level of cell-free DNA (cf-DNA) has been reported in sepsis patients, there has been little direct measurement of circulating free NETs such as myeloperoxidase-conjugated DNA (MPO-DNA). The objectives of this study were to detect NETs in the bloodstream of patients with septic shock, and to assess the correlations of circulating NET levels with organ dysfunction, disease severity, and mortality.

**Methods:**

Fifty-five patients with septic shock admitted to the intensive care units (ICUs) of 35 Japanese hospitals were studied. Septic shock was diagnosed according to the 1997 definition of the American College of Chest Physicians/Society of Critical Care Medicine. To detect circulating NETs, plasma levels of MPO-DNA and cf-DNA were measured by sandwich enzyme-linked immunosorbent assay and by fluorometric assay on days 1, 3, and 7 after the onset of septic shock. Physiological and mortality data were collected from the clinical database.

**Results:**

On days 1, 3, and 7, the patients showed a marked increase in plasma MPO-DNA levels compared with healthy volunteers, whereas the plasma cf-DNA level was only increased significantly on day 1 and then decreased rapidly. A high MPO-DNA level on days 3 and 7 were associated with 28-day mortality.

On days 3 and 7, the MPO-DNA levels were inversely correlated with both the mean arterial pressure and the PaO_2_/F_I_O_2_ ratio, whereas the cf-DNA level was not correlated with either parameter. There was a positive correlation between the plasma MPO-DNA level and the sepsis-related organ failure assessment score on days 3 and 7. Neither cf-DNA nor MPO-DNA levels were correlated with the disseminated intravascular coagulation (DIC) score or the platelet count.

**Conclusion:**

The increase in circulating MPO-DNA in patients with septic shock indicates acceleration of NET formation in the early stages of sepsis. High MPO-DNA levels are associated with the severity of organ dysfunction and 28-day mortality due to septic shock, but not with the DIC score. These results suggest that excessive NET formation contributes to the pathogenesis of septic shock.

**Electronic supplementary material:**

The online version of this article (10.1186/s13054-018-2109-7) contains supplementary material, which is available to authorized users.

## Background

Sepsis is frequently encountered in the intensive care unit (ICU) and has a high mortality rate [1], and so the development of more efficient methods for diagnosis and treatment is required. Methods of assessing the severity of sepsis include the Acute Physiological and Chronic Health Evaluation (APACHE) II score [2] and the Sepsis-Related Organ Failure Assessment (SOFA) score [3]. In addition, numerous biomarkers [4] are frequently used to predict morbidity and mortality in patients with sepsis, including acute-phase proteins such as procalcitonin, C-reactive protein, inflammatory cytokines and chemokines, cell surface proteins of inflammatory cells, and coagulation markers. While these physiological scores and biomarkers are useful, novel biomarkers that can achieve more reliable early diagnosis and assist therapeutic decision making are urgently needed.

Neutrophil extracellular traps (NETs) are a potential biomarker for sepsis because neutrophils are the most abundant of the leukocytes and play a central role in the pathogenesis of sepsis. Neutrophils attack extracellular microbes by releasing toxic proteins and enzymes, including myeloperoxidase (MPO), neutrophil elastase (NE), and defensins, from their granules through the process of degranulation [5].

Recently, detection of DNA fragments circulating in the bloodstream known as cell-free DNA (cf-DNA) has received increasing attention as a prognostic marker. The plasma level of cf-DNA has been examined in various acute and chronic disorders, including trauma [6, 7], sepsis [8], cancer [9], stroke [10], and myocardial infarction [11]. However, plasma cf-DNA levels are not only increased as a result of release from NETs but are also elevated by cellular necrosis and apoptosis. Thus, only detecting an increase in cf-DNA is insufficient evidence to verify NET formation [12].

NET remnants are complexes formed between DNA and neutrophil-derived proteins, including MPO and NE. Myeloperoxidase-conjugated DNA (MPO-DNA) and neutrophil elastase-conjugated DNA (NE-DNA) can be detected in body fluids by enzyme-linked immunosorbent assay (ELISA) as an objective, quantitative, and specific marker of NET formation [13–15]. In this study, circulating levels of soluble NET remnants (MPO-DNA and cf-DNA) were investigated in patients with septic shock to evaluate the extent of NET formation during sepsis.

The aim of this study was to compare the plasma levels of MPO-DNA and cf-DNA between septic shock patients and healthy volunteers, as well as to investigate the relationship of circulating NET levels with 28-day mortality, organ dysfunction, and known parameters of the severity of sepsis.

## Methods

### Patients

The present study was a secondary analysis of a previous prospective observational multicenter cohort study that investigated the incidence and prognosis of septic shock in Japan (Japan Sepsis Study). That study was conducted from April 2008 to March 2010, enrolling patients admitted to the ICUs of 35 Japanese hospitals, among whom 31 patients were eligible for the present study. In addition, collection of medical data and blood samples was continued at the ICU of Aichi Medical University Hospital according to the same study design from April 2011 to July 2017, proving another 24 patients for this study (55 in total). The ethics committee at each hospital approved the protocol of this study, and informed consent was obtained from each patient or from a relative or legal representative if direct consent could not be obtained.

Fifty-five patients with septic shock aged between 36 and 91 years were enrolled who met both of the following criteria: 1) systemic inflammatory response syndrome (SIRS) caused by infection (according to the consensus definition of the American College of Chest Physicians/Society of Critical Care Medicine Consensus Conference Committee [16]); and 2) hypotension (systolic blood pressure ≤ 90 mmHg or decrease in systolic blood pressure ≥ 40 mmHg from baseline) persisting for at least 1 h despite adequate fluid infusion and requiring pressor agents to maintain a systolic blood pressure around 90 mmHg or mean arterial pressure (MAP) < 65 mmHg, associated with hypoperfusion (including lactic acidosis, oliguria, and/or acute changes of mental state) [16]. SIRS was diagnosed if patients had at least two of the following clinical features: temperature < 36 °C or > 38 °C; heart rate > 90/min; respiration rate > 20/min or arterial PCO_2_ < 32 mmHg; and white blood cell count > 12,000/mm^3^ or < 4000/mm^3^, or left shift of the differential white blood cell count with band forms ≥ 10% [17]. The exclusion criteria were terminal cancer, terminal hematological illness, severe hepatic dysfunction (total bilirubin ≥ 10 mg/dl and hepaplastin test ≤ 40%), and refusal to provide written consent. Disseminated intravascular coagulation (DIC) was diagnosed based on the Japanese Association for Acute Medicine DIC diagnosis criteria [18]. All patients were managed according to the Surviving Sepsis Campaign protocol 2008 [19], including fluid resuscitation, use of vasopressors, transfusion, and timely initiation of antibiotic therapy. The following data were obtained from an established registry database: demographic information, site of infection, results of culture and laboratory tests, hemodynamic variables, and therapeutic interventions. All patients were followed up for 28 days after enrollment to assess the 28-day mortality rate. To evaluate organ dysfunction and the severity of sepsis, the SOFA score [3] and the APACHE II score [2] were determined. The control subjects were 13 healthy age-matched volunteers with no significant acute or chronic illnesses.

### Collection of blood samples and testing

Patients provided consent for collection of serial blood samples on days 1, 3, and 7 after the onset of septic shock. The first blood sample was collected within 6 h of the patient meeting the diagnostic criteria for septic shock and was processed within 1 h of collection. Blood samples were also obtained from 13 healthy volunteers. Venous or arterial blood was collected aseptically into heparinized pyrogen-free tubes to determine the plasma levels of MPO-DNA and cf-DNA as well as various cytokines. Each blood sample was centrifuged at 1500 × *g* for 10 min at 4 °C and the plasma thus obtained was frozen at −80 °C for assay of cytokines. Plasma samples for measurement of MPO-DNA and cf-DNA were centrifuged further at 16,000 × *g* for 10 min to remove any residual cells [20].

### Measurement of MPO-DNA

The plasma level of MPO-DNA was measured by ELISA as described previously [14]. In brief, quantitative detection of MPO-DNA was performed using a “sandwich” ELISA with anti-MPO monoclonal antibody (Merck Millipore Corp., catalog no. 07–496) and peroxidase-conjugated anti-DNA monoclonal antibody (Roche Diagnostics, Indianapolis, IN, USA; Cell Death Detection ELISA no. 1154467500: bottle 2). The wells of microtiter strips were coated with a monoclonal antibody specific for MPO to capture MPO-DNA derived from NETs. A peroxidase substrate (2,2’-azino-bis(3-ethylbenzothiazoline-6-sulphonic acid)) was added, which reacted with the bound peroxidase to yield a soluble green product detected at 405 nm. Absorbance readings were proportional to the amount of bound horseradish peroxidase-labeled anti-DNA monoclonal antibody. Results are expressed in arbitrary units. A standard curve for the MPO-DNA assay is shown in Additional file 1: (Figure S1). The optical density (OD) reached a plateau at a sample volume of approximately 50 μl (Additional file 1: Figure S1A, B). Plots of the OD at 405 nm versus sample volume displayed good linearity (*R*^2^ = 0.971) when OD was < 1.5 (Additional file 1: Figure S1B), and thus the MPO-DNA assay was performed within this range.

### Measurement of cf-DNA

The plasma level of cf-DNA was quantified with the Quant-iT PicoGreen dsDNA assay (Life Technologies, CA) according to the manufacturer’s instructions [9]. Briefly, the calf thymus DNA standards (0 to 2 μg/ml) were diluted and incubated for 2 min with Quant-iT PicoGreen reagent at room temperature before measurement of fluorescence to create a standard curve. Samples were compared with the standard curve and results were expressed in ng/ml. Fluorescence intensity (reflecting the DNA content) was measured with a QUBIT® 2.0 Fluorometer (Life Technologies, CA) at 485 nm (excitation) and 538 nm (emission).

### Measurement of interleukin (IL)-6 and IL-8

Plasma levels of IL-6 and IL-8 were measured with ELISA kits (Bio-source, Carlsbad, CA) according to the manufacturer’s instructions.

### Statistical analysis

Data were collected in MS Windows Office Excel 2011. All statistical analyses were performed using SigmaPlot software, version 14 (Systat Software Inc., CA, USA). Categorical variables are reported as absolute values and percentages, while continuous variables are shown as the median with the interquartile range. Repeated measures analysis of variance was used to compare cf-DNA and MPO-DNA levels at three time points (days 1, 3, and 7). Comparisons between paired nonsurvivors and survivors were performed by the Wilcoxon rank sum test. Pearson product-moment correlation coefficient was measured to explore the relationships among the SOFA score, MAP, PaO_2_/F_I_O_2_ (P/F) ratio, DIC score, platelet count, and plasma levels of MPO-DNA and cf-DNA. To determine the discriminative power of MPO-DNA and cf-DNA for 28-day survival, we constructed receiver operating characteristic (ROC) curves and calculated the area under each curve together with its 95% confidence interval (CI).

## Results

### Patient characteristics

The demographic profile and the severity scores calculated in the ICU are summarized in Table 1. All 55 patients fulfilled the criteria for the diagnosis of septic shock, including 29 patients with DIC and 32 patients with adult respiratory distress syndrome (ARDS). Their median age was 68 years, with 71% being men and 29% being women. The 28-day mortality rate was 33%. Among the 55 patients, the source of sepsis was gastrointestinal tract infection in 31, urinary tract infection in 9, respiratory tract infection in 10, and soft tissue infection in 4 patients, while 1 patient had primary bacteremia. Twenty-six patients had undergone surgery before admission to ICU and 8 patients had community/hospital acquired infections. Gram-negative bacilli were isolated from 33 patients, gram-positive cocci were detected in 14 patients, and *Candida albicans* was found in 1 patient. In the remaining 7 patients, no isolates were identified.

**Table 1:** Baseline characteristics of the patients with septic shock and control subjects

Characteristic	Patients with septic shock (*n* = 55)	Control subjects (*n* = 13)	*P* value
Age (years)	68 (55–79)	60 (49–71)	0.47
Male, *n* (%)	39 (71)	8 (62)	0.96
ICU stay (days)	10 (7–18)	n/a	
Site of infection, *n* (%)		n/a	
Abdomen	31 (56)		–
Respiratory tract	10 (18)		–
Urinary tract	9 (16)		–
Skin/Soft tissue	4 (7)		–
Others	1 (2)		–
Community/hospital infections, *n* (%)	8 (15)	n/a	
Pathogen, *n* (%)		n/a	
Gram-positive	14 (27)		
Gram-negative	33 (62)		
Both	7 (13)		
Fungi	1 (2)		
APACHE II score	23 (20–27)	n/a	
SOFA score	12 (9–13)	n/a	
Comorbidities		None	
0, *n* (%)	32 (58)		–
> 1, *n* (%)	23 (42)		
Surgery, *n* (%)	26 (47)	n/a	
DIC, *n* (%)	29 (53)	n/a	
Mechanical ventilation, *n* (%)	47 (85)	n/a	
28-day mortality, *n* (%)	18 (33)	n/a	
Plasma cytokine level (pg/ml)			
IL-6	850 (680–1080)	28 (25–32)	< 0.001
IL-8	380 (308–490)	51 (42–68)	< 0.001

Values are shown as median (interquartile range) unless otherwise stated

The APACHE II score ranges from 0 to 71, with low scores indicating better organ function

The SOFA score ranges from 0 to 24, with lower scores indicating better organ function

*APACHE* Acute Physiology and Chronic Health Evaluation, *DIC* disseminated intravascular coagulation, *ICU* intensive care unit, *IL* interleukin, *n/a* not applicable, *SOFA* Sepsis-Related Organ Failure Assessment

### Plasma cytokine levels

The plasma level of IL-6 was significantly higher in the patients with septic shock than in the healthy controls (Table 1). The plasma IL-8 level was also significantly higher in the sepsis patients than in the controls (Table 1).

### Time course of plasma MPO-DNA and cf-DNA

**Fig. 1:**
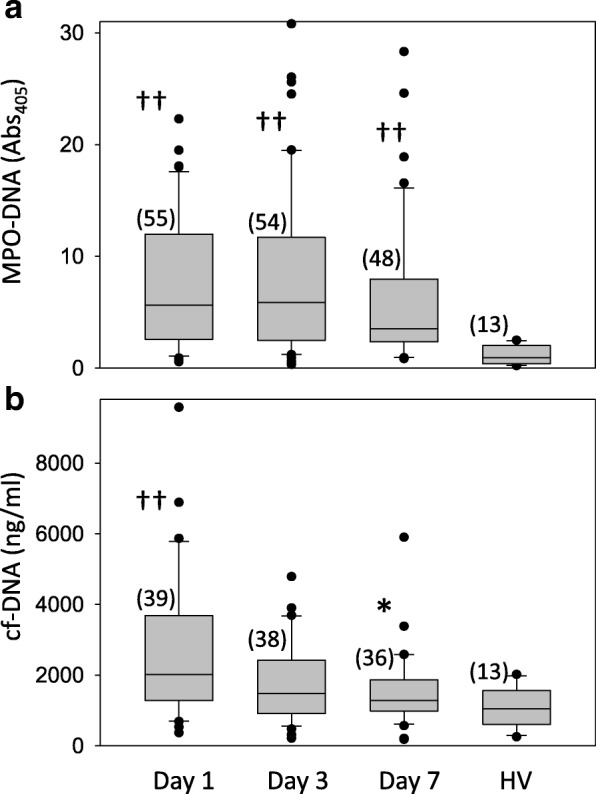
MPO-DNA and cf-DNA levels. Plasma levels of myeloperoxidase (MPO)-DNA and cell-free DNA (cf-DNA) in patients with septic shock and control subjects. MPO-DNA (**a**) and cf-DNA (**b**) levels on day 1, day 3, and day 7 after the diagnosis of septic shock are compared with those in healthy volunteers (HV). Data are shown as box plots with median values (lines inside the boxes), 25th and 75th centiles (borders of the boxes), and whiskers indicating the range. Data beyond the whiskers are plotted as outliers (small circles). The number of patients is shown in parentheses. *p < 0.05 vs. day 1; ^††^*p* < 0.01 vs. healthy volunteers

On days 1, 3, and 7 of septic shock, the plasma MPO-DNA level was significantly higher in the patients compared with the healthy volunteers (Fig. 1). Plasma MPO-DNA remained high until day 7 (Fig. 1). The plasma cf-DNA level on day 1 was significantly higher in the patients compared with the healthy volunteers, but cf-DNA decreased in the patients on day 7 and there was no significant difference from the controls (Fig. 1).

**Fig. 2:**
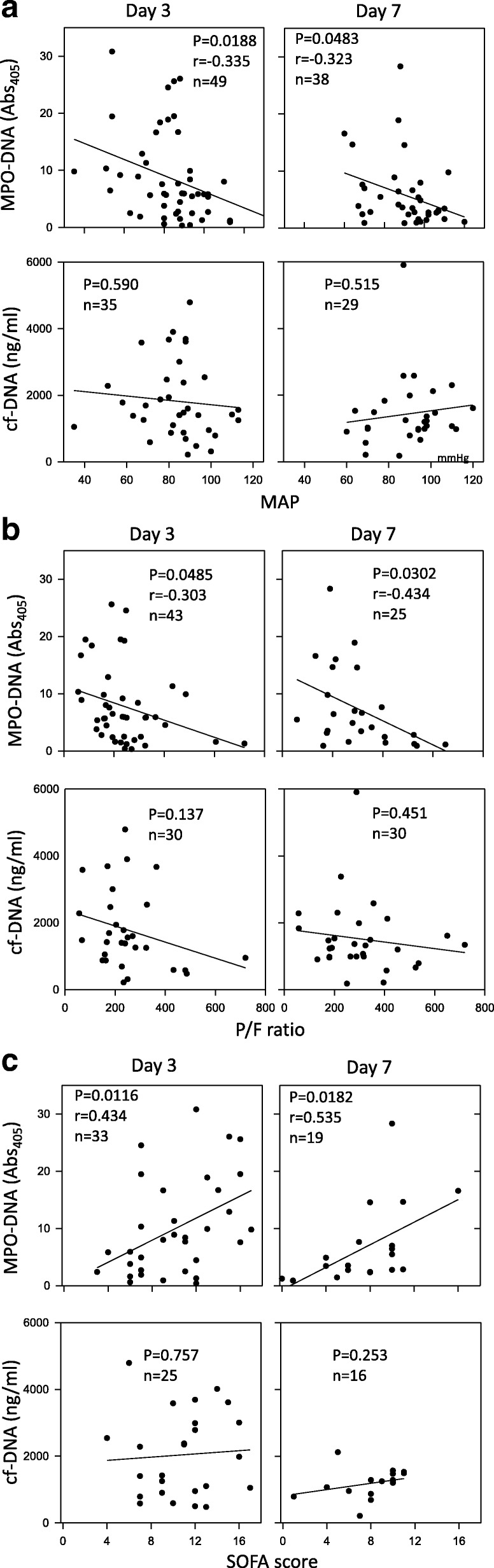
Correlations of MPO-DNA and cf-DNA levels with organ failure parameters. Correlations of myeloperoxidase (MPO)-DNA and cell-free DNA (cf-DNA) levels with the mean arterial pressure (MAP) (**a**), the PaO_2_/F_I_O_2_ (P/F) ratio (**b**), and the Sepsis-Related Organ Failure Assessment (SOFA) score (**c**)

**Fig. 3:**
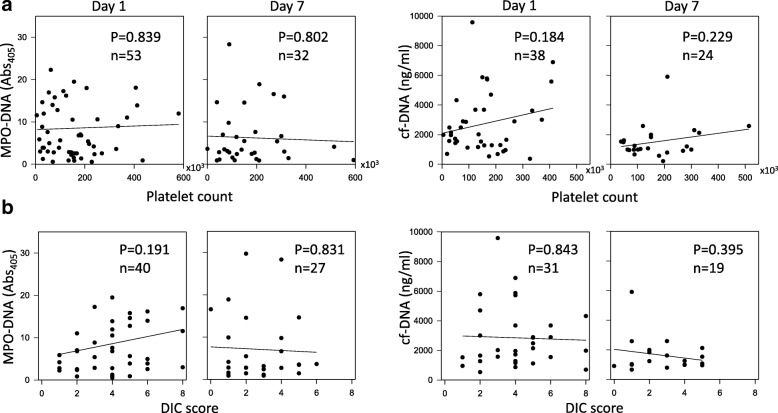
Correlations of MPO-DNA and cf-DNA levels with the platelet count and the DIC score. Correlations of myeloperoxidase (MPO)-DNA and cell-free DNA (cf-DNA) levels with the platelet count (**a**) and the disseminated intravascular coagulation (DIC) score (**b**)

The MPO-DNA level was inversely correlated with both the MAP (Fig. 2a) and the P/F ratio (Fig. 2b) on days 3 and 7 in the patients with sepsis, whereas the cf-DNA level was not correlated with either parameter (Fig. 2a, 2a). In addition, a positive correlation was observed between SOFA score and the plasma MPO-DNA level on days 3 and 7 (Fig. 2c), whereas there was no significant correlation with cf-DNA (Fig. 2c). The cf-DNA and MPO-DNA levels on day 1 were not correlated with MAP, the P/F ratio, or the SOFA score (Additional file 2: Figure S2). In addition, cf-DNA and MPO-DNA levels on days 1, 3, and 7 were not correlated with either the DIC score or the platelet count (Fig. 3 and Additional file 3: Figure S3).

### Prognostic value

**Fig. 4:**
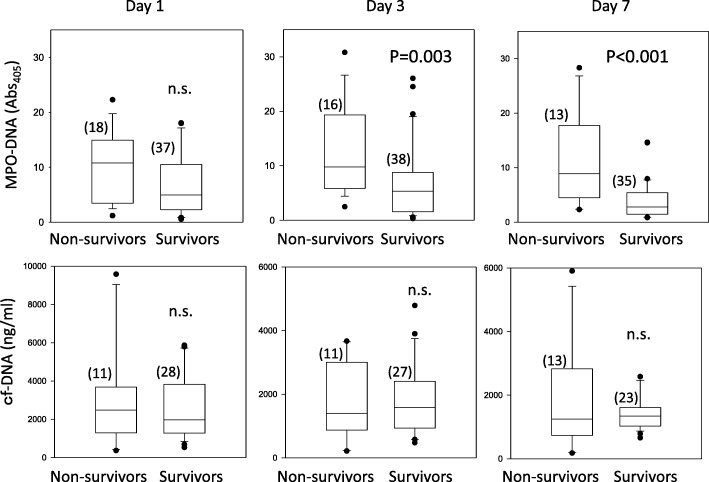
MPO-DNA and cf-DNA levels in nonsurvivors and survivors. Myeloperoxidase (MPO)-DNA and cell-free DNA (cf-DNA) levels in survivors and nonsurvivors with septic shock are shown. The number of patients is shown in parentheses. n.s. not significant

**Fig. 5:**
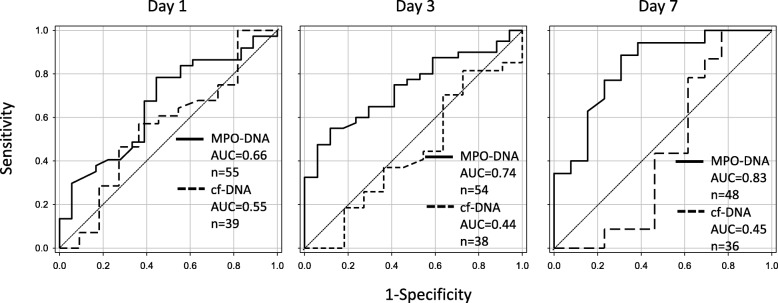
Receiver operating characteristic (ROC) curve analysis. ROC curves for prediction of 28-day mortality by the myeloperoxidase (MPO)-DNA level (solid line) or the cell-free DNA (cf-DNA) level (dashed line) on day 1, day 3, and day 7 after diagnosis of septic shock. The area under the ROC curve (AUC) is shown

A high MPO-DNA level on days 3 and 7 of sepsis was associated with 28-day mortality (Fig. 4). On the other hand, there was no correlation between 28-day mortality and the MPO-DNA level on day 1 or the cf-DNA level at any time (Fig. 4). ROC curves with 95% CIs were obtained for prediction of 28-day mortality using MPO-DNA and cf-DNA levels. Regarding the prediction of 28-day mortality, the area under the ROC curve for MPO-DNA on days 1, 3, and 7 of sepsis was 0.66 (*p* = 0.054, 95% CI 0.51–0.82), 0.74 (*p* = 0.005, 95% CI 0.33–0.77), and 0.83 (*p* = 0.0004, 95% CI 0.70–0.97), respectively (Fig. 5). In addition, the area under the ROC curve for cf-DNA on days 1, 3, and 7 of sepsis was 0.55 (*p* = 0.61, 95% CI 0.51–0.82), 0.44 (*p* = 0.56, 95% CI 0.22–0.65), and 0.45 (*p* = 0.59, 95% CI 0.20–0.69), respectively (Fig. 5).

## Discussion

This study assessed the profile of plasma MPO-DNA and cf-DNA in patients with septic shock, resulting in three main findings. First, both plasma MPO-DNA and cf-DNA levels were significantly higher in septic shock patients on admission than in healthy volunteers. Second, the plasma MPO-DNA level was closely related to the severity of organ dysfunction, including parameters such as the P/F ratio, MAP, and SOFA score, whereas the MPO-DNA level was not related to the DIC score. Third, a high plasma MPO-DNA levels on days 3 and 7 of sepsis were associated with 28-day mortality.

Elevation of the plasma cf-DNA level in our septic shock patients on admission is consistent with previous reports of a several-fold to hundred-fold increase of cf-DNA in septic patients compared with healthy volunteers [8, 21–23]. Plasma cf-DNA has several potential sources in patients with septic shock, including release from NETs, cellular necrosis or apoptosis, and destruction of pathogens [24]. Thus, circulating cf-DNA in septic shock patients is derived from dead cells or from neutrophils that have undergone NETosis. On the other hand, circulating MPO-DNA is more specific for NETs than cf-DNA [25] because these web-like structures released by activated neutrophils are composed of DNA associated with neutrophil granule proteins such as NE, cathepsin G, and MPO [5]. Elevated plasma levels of MPO-DNA have been reported in patients with transfusion-related acute lung injury [26], ANCA-associated small vessel vasculitis [27], severe coronary atherosclerosis [28], and thrombotic microangiopathy [29], and excessive NET formation is related to the pathogenesis of these conditions. Our finding of an increase in MPO-DNA indicates that NET formation is accelerated in the early stage of septic shock. This is consistent with a recent report by Kaufman et al. who measured plasma NE-DNA complexes to assess circulating NETs and identified an increase in patients with sepsis or burns [30]. They concluded that NET formation is augmented in patients with SIRS regardless of whether the inflammatory insult is infectious or traumatic. The elevated plasma levels of IL-6 and IL-8 observed in patients with septic shock in this study may have facilitated NET formation since various cytokines (including tumor necrosis factor (TNF), IL-1, IL-8, and IL-6) are known to accelerate the production of NETs [31, 32].

While this study revealed a significant increase in both cf-DNA and MPO-DNA levels on day 1 of sepsis, the cf-DNA level declined rapidly and MPO-DNA decreased more slowly. The different changes in these two markers over time can be explained by differing levels of resistance to digestion by DNases [33], which selectively digest the DNA threads of NETs. While cf-DNA is rapidly digested by DNases, MPO-DNA appears to be far more stable and consequently persists for longer in the circulation [34]. However, further investigation will be needed to clarify the detailed mechanisms involved in clearance of circulating NET remnants.

There have been some previous reports that the highest plasma cf-DNA levels are observed in septic shock patients who eventually die, suggesting that cf-DNA could be a useful prognostic marker [8, 20–22, 35]. It has also been reported that elevated plasma levels of cf-DNA predict the development of multiple organ dysfunction in trauma patients [7] and septic patients [8]. However, we found no correlation between the plasma cf-DNA level and mortality, with MPO-DNA being superior to cf-DNA as a prognostic marker in patients with septic shock. The plasma MPO-DNA level was also closely related to several parameters of organ dysfunction, including the P/F ratio, MAP, and SOFA score. These differences in the relation with organ dysfunction or mortality between MPO-DNA and cf-DNA can be explained by the differing specificity of these two markers for NETs. Hamaguchi et al. studied a mouse model of sepsis due to cecal ligation and puncture (CLP), reporting that plasma cf-DNA is not derived from NETs released by activated neutrophils and is mainly from other host cells [36]. In addition, a study performed in sepsis patients showed that cf-DNA predominantly has a low molecular weight (150–250 bp) corresponding to the size of apoptotic nucleosomal DNA [23]. Thus, an increase in cf-DNA is probably related to cellular damage and apoptosis that may occur in various tissues by several mechanisms. Moreover, the influence of impaired renal and hepatic function on clearance of cf-DNA seems to be complex [35]. Taken together, further prospective studies are needed to clarify the origin of cf-DNA and its usefulness as an accurate and sensitive biomarker for sepsis.

At present, it is unclear whether NET formation has an essential role in host defenses against bacterial invasion [37]. In a mouse CLP model, Czaikoski et al. showed that degradation of circulating DNA (a major constituent of NETs) by systemic treatment with recombinant human DNase (rhDNAse) led to earlier death than in the control group, possibly due to an increase in the bacterial load [38]. They concluded that NETs have a beneficial role in killing pathogens and that depletion of NETs leads to aggravation of polymicrobial sepsis. On the other hand, it has been reported that NETs are not required to control bacterial proliferation because congenital absence of NETs in peptidylarginine deiminase 4 knockout mice or treatment with rhDNAse to prevent NET formation did not increase the bacterial load in animals with sepsis [39, 40]. Lefrançais et al. showed that an increase in NETs, as assessed from the plasma level of NE-DNA complexes, was associated with the severity of ARDS, while lower plasma DNase I levels were associated with development of sepsis-induced ARDS [15]. They concluded that strategies to reduce NET levels could have a favorable effect on lung function. The inverse correlation between the plasma NET level and the P/F ratio observed in the present study corresponds to the findings reported by Lefrançais. Although an essential role of NETs in killing pathogens has not been demonstrated at present, our results confirm that excessive NET formation contributes to the development of multiple organ dysfunction and mortality in patients with septic shock.

According to the immunothrombosis hypothesis, NET formation is linked to platelet aggregation and hypercoagulation associated with septic shock [41]. NETs can support immunothrombosis through binding to von Willebrand factor (vWF) [42] and by activation of the platelet Toll-like receptor (TLR)4-dependent interaction between platelets and neutrophils [43]. However, we found no correlation between the DIC score and the plasma MPO-DNA level in the present study. Consistent with our result, Kaufman et al. reported that neither platelet TLR4 nor plasma vWF levels were correlated with plasma NE-DNA in sepsis patients, suggesting that these two factors are not involved in the acceleration of NETosis in this condition [30]. Very recently, Delabranche et al. showed that circulating NET levels (assessed by measuring plasma MPO-DNA) are elevated in sepsis patients with DIC compared with sepsis patients without DIC [44], and they suggested that NETs may play a critical role in the onset of DIC accompanying sepsis. The discrepancies between these studies may be attributable to differences in the methods of measuring circulating NETs, as well as differences in the patients enrolled, statistical analysis, and treatment (including anticoagulant therapy for DIC). Anticoagulant therapy, including antithrombin, recombinant thrombomodulin (rTM), and protease inhibitor supplementation, is strongly recommended in the Japanese guidelines for management of DIC [45, 46], and these therapies may affect the formation of NETs. Recently, administration of antithrombin was reported to reduce NET formation in the lungs during lipopolysaccharide (LPS)-induced endotoxemia [47]. Serine protease inhibitors [48] and rTM [49] are also known to inhibit formation of NETs in vitro.

Some limitations of the present study should be considered. We only examined a limited number of patients, which means that our results require validation on a larger scale. Another limitation is that our age-matched control group consisted of healthy volunteers and not critically ill patients with or without sepsis. Therefore, our findings may have been due to acute illness or underlying comorbidities and not necessarily related to septic shock per se.

## Conclusions

We demonstrate that the plasma MPO-DNA level is significantly increased in septic shock. The MPO-DNA level was closely related to 28-day mortality and to markers of the severity of organ dysfunction, including the P/F ratio, MAP, and SOFA score. These findings support the concept that NET formation is accelerated in sepsis and contributes to the pathogenesis of septic shock. Further investigation will be required to clarify the role of NETs in DIC. It is possible that development of therapeutic interventions targeting NETs may be worthwhile for preventing organ dysfunction and improving the outcomes of sepsis. Finally, MPO-DNA is a sensitive and accurate marker of septic shock that can be tested rapidly and noninvasively.

## Additional files

Additional file 1: **Figure S1.** Determination of linearity of the MPO-DNA assay. The linear range of optical density (OD) in the MPO-DNA assay was determined with various sample volumes: A) 0, 6.25, 12.5, 25, 50, 75, and 100 μl; B) 0, 6.25, 12.5, 25, and 50 μl (*n* = 4 for each sample volume). (PPTX 64 kb)

Additional file 2: **Figure S2.** Correlations of MPO-DNA and cf-DNA levels with organ failure parameters. Correlations of MPO-DNA and cf-DNA levels with the MAP (A), the P/F ratio (B), and the SOFA score (C) on day 1 after the diagnosis of septic shock. (PPTX 114 kb)

Additional file 3: **Figure S3.** Correlations of MPO-DNA and cf-DNA levels with the platelet count and the DIC score. Correlations of MPO-DNA and cf-DNA levels with the platelet count (A) and the DIC score (B) on day 3 after the diagnosis of septic shock. (PPTX 76 kb)

## Abbreviations

APACHE: Acute Physiological and Chronic Health Evaluation
ARDS: Adult respiratory distress syndrome
cf-DNA: Cell-free DNA
CI: Confidence interval
CLP: Cecal ligation and puncture
DIC: Disseminated intravascular coagulation
ELISA: Enzyme-linked immunosorbent assay
ICU: Intensive care unit
IL: Interleukin
MAP: Mean arterial pressure
MPO: Myeloperoxidase
NE: Neutrophil elastase
NET: Neutrophil extracellular trap
OD: Optical density
P/F: PaO_2_/F_I_O_2_
rhDNase: Recombinant human DNase
ROC: Receiver operating characteristic
rTM: Recombinant thrombomodulin
SIRS: Systemic inflammatory response syndrome
SOFA: Sepsis-Related Organ Failure Assessment
TLR: Toll-like receptor
vWF: Von Willebrand factor
