# Supplementary figures and images for: Plasma myeloperoxidase-conjugated DNA level predicts outcomes and organ dysfunction in patients with septic shock
Source: Crit Care. 2018 Jul 13;22:176. doi: 10.1186/s13054-018-2109-7 (PMC6045839; doi:10.1186/s13054-018-2109-7)

## Slide 1
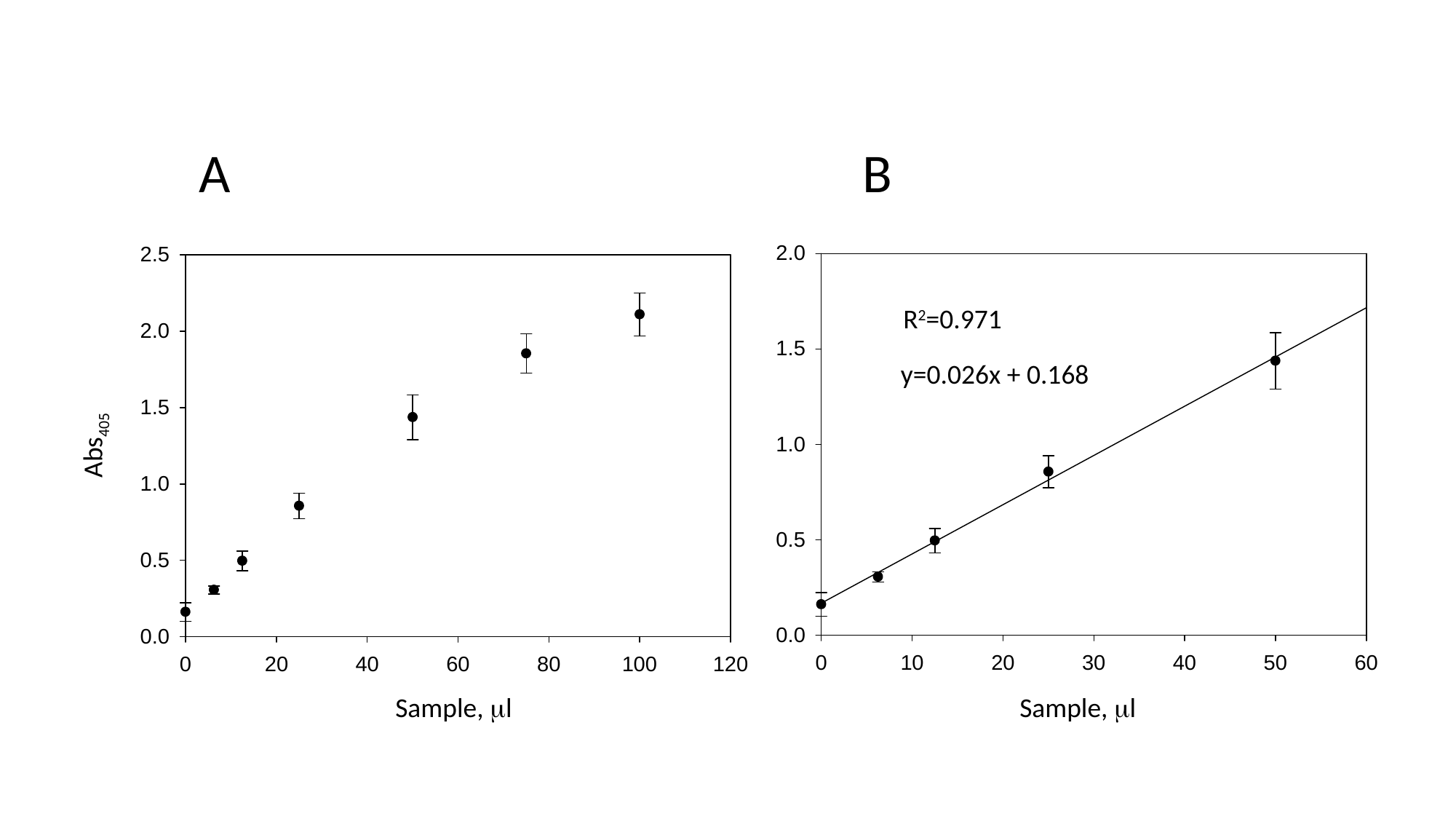

A
B
R2=0.971
y=0.026x + 0.168
Abs405
Sample, ml
Sample, ml

Supplement: Supplementary file 1 — Figure S1. Determination of linearity of the MPO-DNA assay. The linear range of optical density (OD) in the MPO-DNA assay was determined with various sample volumes: A) 0, 6.25, 12.5, 25, 50, 75, and 100 μl; B) 0, 6.25, 12.5, 25, and 50 μl (n = 4 for each sample volume). (PPTX 64 kb) [file 13054_2018_2109_MOESM1_ESM.pptx]
