# Supplementary material for: Plasma myeloperoxidase-conjugated DNA level predicts outcomes and organ dysfunction in patients with septic shock
Source: Crit Care. 2018 Jul 13;22:176. doi: 10.1186/s13054-018-2109-7 (PMC6045839; doi:10.1186/s13054-018-2109-7)
Supplement: Supplementary file 2 — Figure S2. Correlations of MPO-DNA and cf-DNA levels with organ failure parameters. Correlations of MPO-DNA and cf-DNA levels with the MAP (A), the P/F ratio (B), and the SOFA score (C) on day 1 after the diagnosis of septic shock. (PPTX 114 kb) [file 13054_2018_2109_MOESM2_ESM.pptx]

## Slide 1
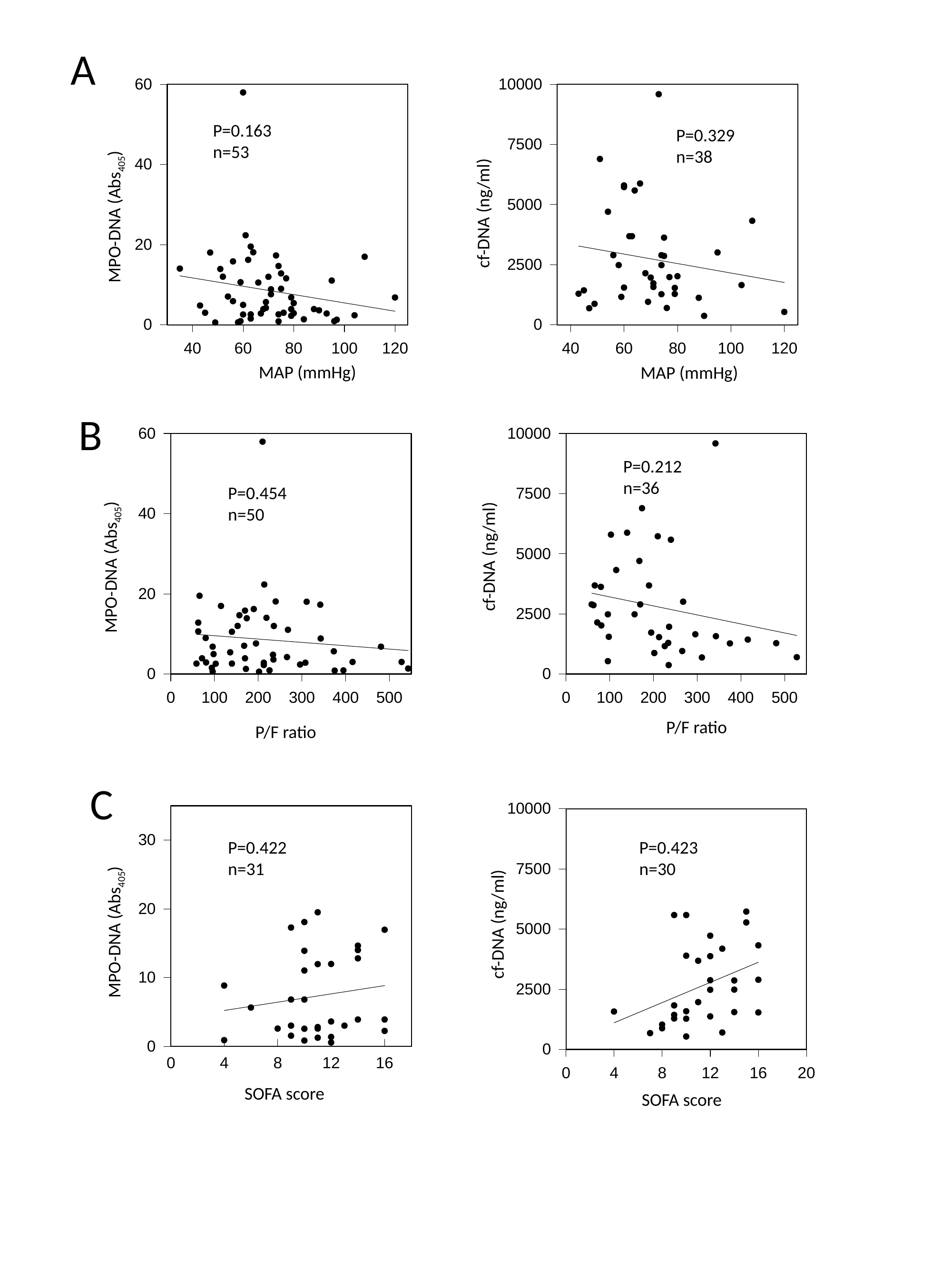

A
P=0.163
n=53
P=0.329
n=38
cf-DNA (ng/ml)
MPO-DNA (Abs405)
MAP (mmHg)
MAP (mmHg)
B
P=0.212
n=36
P=0.454
n=50
cf-DNA (ng/ml)
MPO-DNA (Abs405)
P/F ratio
P/F ratio
C
P=0.422
n=31
P=0.423
n=30
cf-DNA (ng/ml)
MPO-DNA (Abs405)
SOFA score
SOFA score
