# Supplementary material for: Plasma myeloperoxidase-conjugated DNA level predicts outcomes and organ dysfunction in patients with septic shock
Source: Crit Care. 2018 Jul 13;22:176. doi: 10.1186/s13054-018-2109-7 (PMC6045839; doi:10.1186/s13054-018-2109-7)
Supplement: Supplementary file 3 — Figure S3. Correlations of MPO-DNA and cf-DNA levels with the platelet count and the DIC score. Correlations of MPO-DNA and cf-DNA levels with the platelet count (A) and the DIC score (B) on day 3 after the diagnosis of septic shock. (PPTX 76 kb) [file 13054_2018_2109_MOESM3_ESM.pptx]

## Slide 1
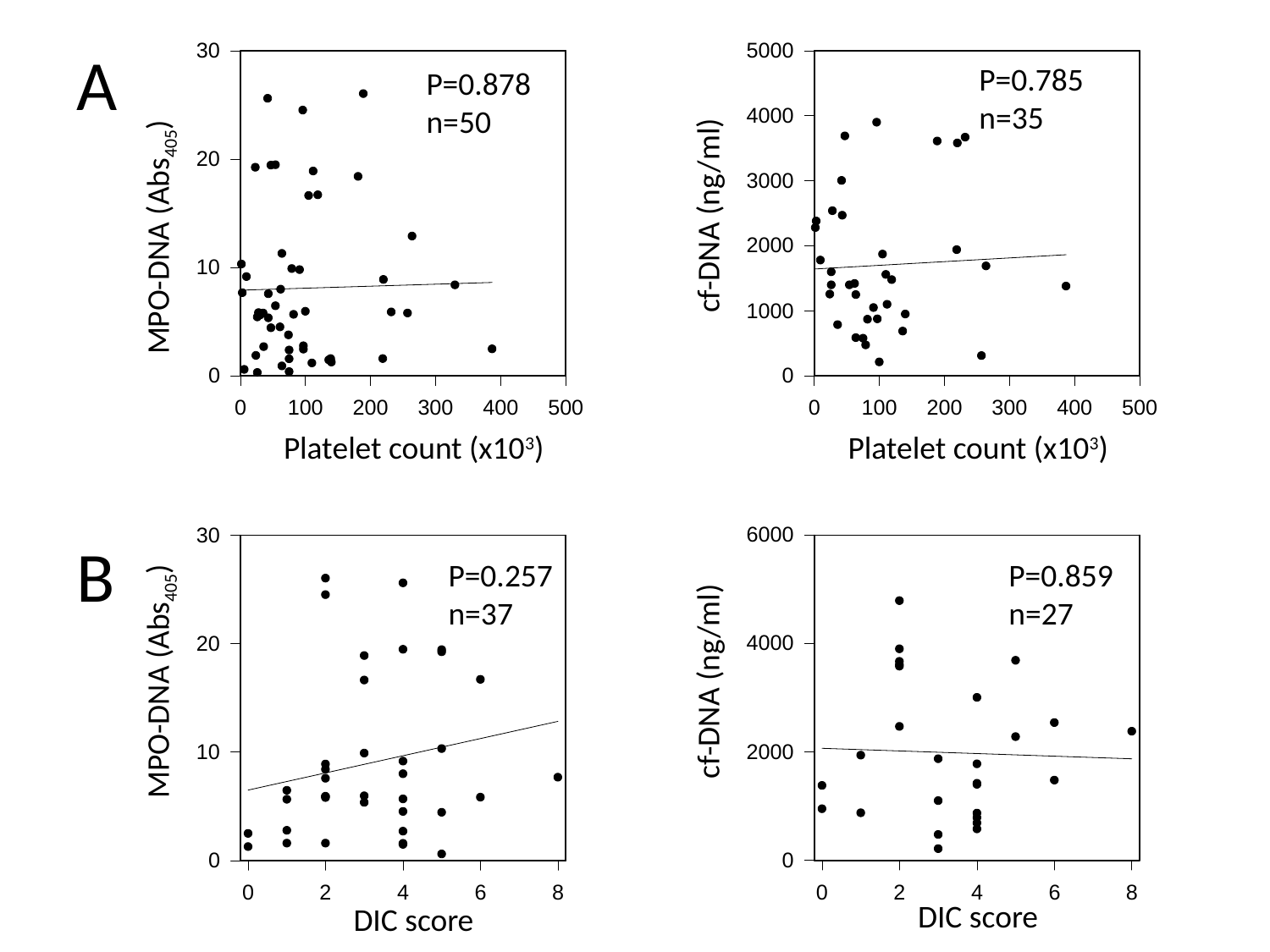

A
P=0.785
n=35
P=0.878
n=50
cf-DNA (ng/ml)
MPO-DNA (Abs405)
Platelet count (x103)
Platelet count (x103)
B
P=0.257
n=37
P=0.859
n=27
cf-DNA (ng/ml)
MPO-DNA (Abs405)
DIC score
DIC score
